# Schistosomiasis diagnosis: Challenges and opportunities for elimination

**DOI:** 10.1371/journal.pntd.0012282

**Published:** 2024-07-11

**Authors:** Ombeni Ally, Bernard N. Kanoi, Lucy Ochola, Steven Ger Nyanjom, Clement Shiluli, Gerald Misinzo, Jesse Gitaka

**Affiliations:** 1 Department of Molecular Biology and Biotechnology, Pan African University Institute for Basic Sciences, Technology and Innovation (PAUSTI), Nairobi, Kenya; 2 Department of Biology, College of Natural and Mathematical Sciences, University of Dodoma, Dodoma, Tanzania; 3 Centre for Research in Infectious Diseases, College of Graduate Studies and Research, Mount Kenya University, General Kago Rd, Thika, Kenya; 4 Department of Tropical and Infectious Diseases, Institute of Primate Research, Nairobi, Kenya; 5 Department of Biochemistry, Jomo Kenyatta University of Agriculture and Technology, Nairobi, Kenya; 6 SACIDS Africa Center of Excellence for Infectious Diseases, Sokoine University of Agriculture, Morogoro, Tanzania; Emory University School of Medicine Credentialed through Emory Healthcare, UNITED STATES

## Abstract

**Overview:**

The roadmap adopted by the World Health Organization (WHO) for eliminating neglected tropical diseases aims to eliminate schistosomiasis, as a public health concern, by 2030. While progress has been made towards reducing schistosomiasis morbidity control in several sub-Saharan African countries, there is still more that needs to be done. Proper surveillance using accurate diagnostics with acceptable sensitivity and specificity is essential for evaluating the success of all efforts against schistosomiasis. Microscopy, despite its low sensitivity, remains the gold standard approach for diagnosing the disease. Although many efforts have been made to develop new diagnostics based on circulating parasite proteins, genetic markers, schistosome egg morphology, and their paramagnetic properties, none has been robust enough to replace microscopy. This review highlights common diagnostic approaches for detecting schistosomiasis in field and clinical settings, major challenges, and provides new and novel opportunities and diagnosis pathways that will be critical in supporting elimination of schistosomiasis.

**Methods:**

We searched for relevant and reliable published literature from PubMed, Scopus, google scholar, and Web of science. The search strategies were primarily determined by subtopic, and hence the following words were used (schistosom*, diagnosis, Kato–Katz, antibody test, circulating antigen, POC-CCA, UCP-LF-CAA, molecular diagnostics, nucleic acid amplification test, microfluidics, lab-on a disk, lab-on chip, recombinase polymerase amplification (RPA), LAMP, portable sequencer, nanobody test, identical multi-repeat sequences, diagnostic TPPs, REASSURED, extraction free), and Boolean operators AND and/OR were used to refine the searching capacity. Due to the global public health nature of schistosomiasis, we also searched for reliable documents, reports, and research papers published by international health organizations, World Health Organization (WHO), and Center for Disease control and Elimination.

## Introduction

The global prevalence of schistosomiasis remains too high, with a great burden being carried by sub-Saharan Africa [1,2]. According to World Health Organization (WHO), about 240 million people from 78 countries worldwide required preventive chemotherapy for schistosomiasis in 2020 [2,3], over 90% of whom live in Africa [1,3]. There are 2 major forms of schistosomiasis; intestinal, which is mainly caused by *Schistosoma mansoni*, *S*. *mekongi*, *S*. *japonicum*, *S*. *intercalatum*, and *S*. *guineensis*, and urogenital, caused by *S*. *haematobium* [3]. Of the 6 *Schistosoma* species, *S*. *mansoni* and *S*. *haematobium* remain the most prevalent [1]. *S*. *mansoni* causes liver inflammation, and undernutrition and growth retardation in children, while *S*. *haematobium* causes haematuria, painful and frequent urination, urinary tract infections, and in severe cases, bladder and kidney damage, and high infertility rates in regions with a high prevalence of schistosomiasis [1,4].

Schistosomiasis is a preventable and treatable disease [2,5], and sensitive diagnostics would enable early intervention, saving many lives, particularly those of small-scale agricultural and fishing communities. In the fight against neglected tropical diseases, many efforts have been made to eliminate schistosomiasis as one of the major public health concerns [1,3,6–8], and in 2022, the WHO updated guidelines for controlling and eliminating human schistosomiasis [3]. The WHO recommends an integrated approach, combining the extension of preventive chemotherapy to all people at risk from 2 years of age in communities with a ≥10% prevalence, sanitation, treatment in health facilities, and snail control to eliminate schistosomiasis as a public health problem. Treatment campaigns have been scaled-up in several sub-Saharan African countries where most people at risk live [1,8]. The evaluation of mass drug treatment campaigns conducted in 2022 indicated a significant reduction of schistosomiasis in Burundi, Eritrea, Eswatini, Gambia, Lesotho, and Rwanda [8].

Diagnosis plays a critical role in measuring the impact of interventions as it determines levels of reduction of disease, infections, and parasite transmission. The sensitive, affordable, and accurate diagnostics are necessary to monitor, treat, and perform continuous surveillance of schistosomiasis cases [2,3,9]. Efforts have been made to develop new diagnostic tools for schistosomiasis, but sensitivity, specificity, and affordability remain the major challenges [2,3,9].

In October 2019, WHO established a diagnostic technical advisory group (DTAG) to develop target product profiles (TPPs) for better diagnostic tests for neglected tropical diseases, including schistosomiasis, as most existing tests are inadequate [9]. The diagnostic target set by WHO requires a highly sensitive point of care diagnostics specific to *S*. *mansoni* or *S*. *haematobium*, and *S*. *mansoni* and *S*. *haematobium*, as acceptable and preferred criteria, respectively [3,9]. The test should also enable point-of-care (POC) detection with minimum infrastructure for a single field visit decision. Ideally, the POC should cost as less as microscopy (maximum of $3 per sample) in contrast to most laboratory-based tests, whose initial capital could be as high as $10,000 [9].

**Fig 1:**
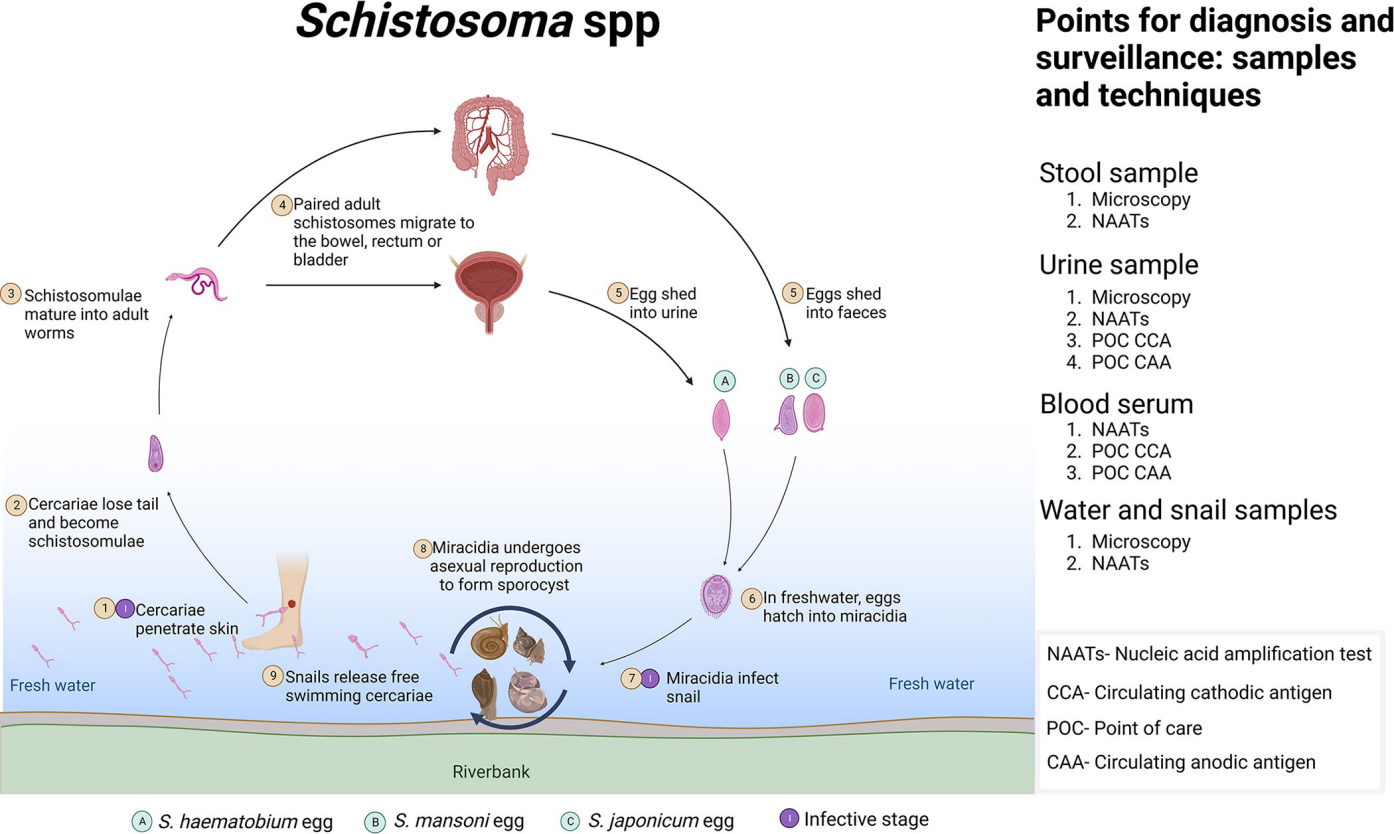
Showing the relationship between the life cycle of schistosomes and diagnostic approaches. Image created with BioRender.com.

Diagnosis of schistosomiasis targets different stages of the parasites complex lifecycle as illustrated in Fig 1. In general, the diagnosis of schistosomiasis is routinely carried out using microscopy, which though affordable and highly specific, is less sensitive [9–12]. Recently, antibody-based assays, parasite antigen detection, and nucleic acid amplification assays have been developed [13–15], but none is robust enough to replace microscopy as the gold standard [3]. Therefore, there is an urgent need to develop rapid and affordable POC diagnostics that are highly sensitive, particularly in sub-Saharan Africa, where the disease is still a major public health burden.

### Common approaches for diagnosing schistosomiasis in field and clinical settings

#### Microscopic examination of schistosome eggs

In endemic countries, the diagnosis of schistosomiasis commonly relies on identifying schistosome eggs (Table 1). This detection method involves utilizing Kato–Katz (KK) and urine filtration techniques to prepare stool and urine samples, respectively. Subsequently, the samples undergo microscopic examination to detect intestinal and urogenital schistosomiasis [11,13,16].

**Table 1:** A summary of common approaches for diagnosing schistosomiasis.

Assay	Advantages	Challenges	References
Microscopy	• Estimate both prevalence and intensity of infection • Cost effective • Highly specific	• Poor sensitivity • Require microscope and trained personnel	[9,10,17]
Point of care targeting circulating cathodic antigen (POC-CCA)	• Estimate both prevalence and intensity of infection • More sensitive than microscopy • Commercially available	• Variable performance on different batches of POC-CCA • High false positivity • Much more effective on detecting *S*. *mansoni* than other species	[9,11,18,19]
Antibody test	• Sensitive than microscopy • Can be multiplexed with other serological assays	• Unable to distinguish active from former infection	[9,14,15]
Conventional PCR (cPCR)	• Can be multiplexed • More sensitive than microscopy • Cost effective than qPCR	• Require expensive machines and trained personnel • Post PCR processing increases the chance of contamination	[7,20]
Real time quantitative PCR (qPCR)	• Does not require post PCR processing • Higher sensitive than cPCR • Can be multiplexed • Specificity can be improved using probe	• The requirement for triplicate reaction makes qPCR highly expensive • Require expensive machines and trained personnel	[12,20,21]
Loop-mediated isothermal amplification (LAMP)	• Cost effective compared to cPCR and qPCR • Combination of LAMP and lateral flow device reduces the risk of contamination	• The complicated initial optimization • Prone to false positive due to carryover contamination	[22,23]

Microscopy remains the gold standard, mainly due to its high specificity compared to other diagnostic approaches, and the WHO recommends its use in individuals with medium to high parasite load for consistent results [2,3]. In individuals with low infection, the number of schistosome eggs is sometimes very low and difficult to detect [24]. In addition, the availability of eggs in the urine and stool is affected by several factors that include, diet, the intensity of infection, time for sample collection, and the consistency of stool sample [16,25], which cumulatively and significantly affects the accuracy of microscopic examination.

#### Detection of circulating proteins

Adult schistosomes and their eggs contain specific proteins that can be detected in blood circulation, urine, stool, and saliva [11,26]. Antigen-based assays have been developed and evaluated, targeting the circulating anodic antigen (CAA) and circulating cathodic antigen (CCA) [18,27–30]. The CCA and CAA are regurgitated from the gut of schistosomes, excreted in the patient’s urine, and positive results indicate an active infection. The POC targeting CCA is commercially available; however, there are concerns over lack of sensitivity and specificity, caused by reported discrepancies when the assay was evaluated against confirmed *Schistosoma*-positive and negative samples [3,18,28,31]. One study reported that POC-CCA have high false positives in pregnant women (32.7%) and pre-school-aged children (46.5%) [19]. In addition, significant variation in sensitivity and specificity among different batches and versions have been reported, and frequent fluctuations in assay replicates have also been reported [18,28,29], imposing some questions to the current manufacturing process of a POC-CCA test.

*Schistosoma* infections may also induce the production of antibodies, which can be used as diagnostic markers. The detection of immunoglobin G (IgG) and immunoglobin M (IgM), which are produced by the body in response to the presence of cercaria secretions, schistosome egg antigens, or soluble adult antigens, are the most commonly used antibody-based methods [2,6,14,15]. Antibody-based methods are also highly sensitive compared to the microscopy approach; hence, they have been recommended for individuals with low infection intensity. The major challenge for antibody-based methods is that they are associated with false positive due to the continuous circulation of antibodies after the infection has been cleared [3,25]. Secondly, the level of antibodies does not necessarily indicate the level of parasite intensity, and there might be an occurrence of cross-reactivity since schistosomes share antigen epitopes with some helminths such as *Filaria* spp., *Echinococcus* spp., and *Strongyloides* [15].

#### Molecular-based diagnostics

Nucleic acid amplification tests use specific nucleotides targeting specific location(s) of the schistosome, hence enabling the amplification of the targeted region(s). Various nucleic acid amplification tests (NAATs) have been developed and evaluated to diagnose schistosomiasis [7,12,25,32,33]. There are 3 commonly used NAATs for detecting schistosomiasis in field and clinical settings [7]: conventional PCR, real-time PCR, and loop-mediated isothermal amplification assay (LAMP). Other NAATs-based assays include digital droplet-PCR, nested-PCR, and recombinase polymerase amplification (RPA) [7]. NAATs usually demonstrate much better sensitivity than microscopy, making them suitable for low infection burden [7,12,25].

Different PCR-based assays have been developed to target specific locations in the *Schistosoma* genome, including the ribosomal subunits 18S rDNA, SjR2 retrotransposon, SM1-7 tandem repeat sequence, Dra1 repeat sequence, cytochrome c oxidase-COX I, 28s rDNA, and SSU-rRNA, mitochondrial genes, and the internal transcriber-spacer-2 sequence–ITS2 [6,7,25]. The Sm1-7 tandem repeats and Dra-1 repeat sequences have been targeted for an improved sensitivity in diagnosing *S*. *mansoni* and *S*. *haematobium* infection, respectively [34].

Real-time PCR allows the detection of schistosomes in real-time, and a multiplex version is available [12,35]. Real-time PCR offers higher sensitivity than conventional PCR and can indicate the infection intensity of an infected individual, but the major limitation of the PCR-based methods is caused by cumbersome DNA extraction and purification procedures and the need for sophisticated tools and experts [7].

Loop-mediated isothermal amplification (LAMP) allows simple and rapid detection without the requirement for expensive equipment such as a thermocycler and automated documentation systems [32,36,37]. In 2018, LAMP was evaluated for its potential to diagnose schistosomiasis in low transmission settings, and results indicated that LAMP was more efficient in detecting *S*. *mansoni* compared to KK in human samples and nested-PCR in snails [33], and Brazil and China have already employed the use of LAMP for snail surveillance [33]. The method has also been used for field monitoring of snails infected with *S*. *mansoni*, *S*. *haematobium*, and *S*. *japonicum*, and studies have also demonstrated that LAMP can detect schistosome DNA from human stool, urine, blood serum, and snail samples [22,36,38]. The use of specific inner and outer sets of primers makes LAMP assay highly specific in detecting the specific target of schistosome parasites. However, the use of multiple primers complicates the initial optimization of LAMP assays, and also if good laboratory practices are not followed carefully, aerosols with high concentrations of LAMP amplified products may easily be formed while handling reaction tubes, leading to contamination of the surrounding area. Subsequently, carryover contamination may occur through contaminated reagents, pipettes, working surfaces, or personal protective gear [20,23].

### Major challenges in diagnosing schistosomiasis

#### The need for a genus-specific POC test

Regardless of the causative agents, schistosomiasis treatment is based on using a single drug (Praziquantel), at least to date [2,3,8]. Researchers have been able to come up with multiplex-based PCR assay [12,35] but the need for DNA extraction, cold chains, and sophisticated machines such as thermocyclers are the 3 major challenges to its use, especially in remote settings areas, where the disease affects the most. CCA and CAA have been investigated as genus-specific biomarkers to detect schistosomes, and POC diagnostics based on the 2 antigens are available [29–31]. The POC-CCA test, which is commercially available, can accurately demonstrate moderate to heavy *S*. *mansoni* infection, and some studies have indicated that it cannot be used in low infection settings [28,39].

The other potential POC diagnostic is up-converting phosphor-lateral flow (UCP-LF)-CAA test, which has indicated much more sensitivity than microscopy and qPCR, and the current version allows for dry reagent formats for easy transport and storage at ambient temperature [13,30,40]. Still, the potential for its use as a POC test in its current format is limited as it is laboratory-based and requires trained personnel and centrifugation equipment [40]. CCA and CAA are also inappropriate biomarkers for detecting prepatent infection, and hence, their uses in surveillance studies for mass drug administration programs are restricted, especially where there is low transmission. Therefore, for elimination purposes, it is clear that there is a need to validate or develop and make publicly available an affordable and sensitive POC diagnostic that can simultaneously detect multiple schistosomes.

#### Sensitivity and specificity issues for the common diagnostic tools

The debate about the low sensitivity of microscopy has existed for several decades, and the WHO recommends much more sensitive approaches to diagnose schistosomiasis in low-transmission settings [9]. Although NAATs, POC-CCA, and antibody tests are reported to offer much better sensitivity than microscopy, studies have indicated a potential risk for these assays to designate negative for some positive samples, which might significantly affect the actual prevalence estimates and elimination strategies, particularly where *Schistosoma* infection burden is low [2,10,12,17].

According to the WHO, the overall performance of *Schistosoma* infection diagnosing tools remains moderate, with most tools having low to moderate sensitivity or specificity [2]. The report also called for further evaluation of immunological and molecular tests, as there is no enough data to make an informed decision on the performance of most diagnostics [2]. In contrast, the recently published systematic review and meta-analysis by Feleke and colleagues [17] evaluated the performance of PCR and ELISA, among other tests. Still, the conclusion made by the authors might need further analysis. For example, the authors performed a meta-analysis by pooling different PCR that target different genes or target(s) (single or multiple repeat targets), with variation in source of samples (urine or stool) or reference tests (urine examination or KK), which is likely to invalidate the estimation of the accuracy of the performance of PCR tests [17].

Few studies, however, have reported that the sensitivity of NAATs for *Schistosoma*, including qPCR, depends on the intensity of infection [12,41–43]. A study to compare the KK to *Schistosoma*-specific ITS2 qPCR found that percentages of PCR positives varied from 79% to 87% in the group with light-intensity infections (1 to 99 eggs per gram of feces (epg)), 83% to 97% in the moderate egg count group (100 to 399 epg), and 100% for heavy infections (≥400 epg) [12]. In another comparison, Keller and colleagues [44] reported that real-time PCR targeting the Dra1 repeat unit in *S*. *haematobium* resulted in an overall sensitivity of 89.5% with 82.8% specificity, missing 11 out of 105 urine samples with *S*. *haematobium* eggs, but the sensitivity increased to 96.4% (27/28) for ≥50 epg samples. Recent findings on isothermal amplification assays, including LAMP and RPA have also indicated a potential to offer comparable sensitivity to much more than PCR, and this shows their potential to replace PCR, not only based on their cost effectiveness but also sensitivity [33,36,45].

Protein-based biomarkers (CCA, CAA, and antibodies) are also ubiquitous targets for *Schistosoma* infection, with POC-CCA being WHO recommended for surveillance of *S*. *mansoni* [2]. When qPCR was compared to CCA and CAA-based tests, Armoo and colleagues [31] and Hoekstra and colleagues [10] reported reduced sensitivity of qPCR compared to urine CCA assay and (UCP-LF) CAA test, respectively. Nevertheless, (UCP-LF) CAA is not commercially available, and the reported variation in sensitivities and specificities between different versions and batches of the commercially available POC-CCA tests limits their reliability in diagnosing *Schistosoma* infection [18,29,46]. Graeff-Teixeira and colleagues [18] concluded, quoted, “*the indicated limitations of specificity and reproducibility with POC–CCA prevents an unrestricted recommendation for its application not only as a cut-off point for MDA scheme but also as a reliable diagnostic tool for selective chemotherapy in low endemic areas and at final stages of transmission interruption*,*”* highlighting the need for manufacturers to standardize the production, assuring quality and reproducibility of POC-CCA tests.

Few studies have also reported that antibody-based tests offer much better sensitivity than microscopy [13–15]. Still, their specificity is much more reduced in communities where *Schistosoma* infection is co-infected with other related helminths or the availability of the circulating antibodies in some successfully treated individuals [9,13]. For instance, Hoermann and colleagues [15] reported the *Schistosoma* ICT IgG-IgM rapid diagnostic test to have 100% sensitivity in detecting single or multiple schistosomes, and the test indicated 100% specificity in serum from healthy individuals, while samples with helminths related to schistosomes lowered specificity to 85%. Discrepancies in the performance of the existing tests and the insufficient data to support the feasibility of using any new diagnostics beyond the conventional tools for diagnosis, KK, POC-CCA, and urine microscopy [2], make an open call for developing new diagnostic approaches for schistosome infections and evaluating existing ones.

#### Mis-diagnosis of male and female genital schistosomiasis

In 2015, the WHO published a pocket atlas for clinical healthcare professionals, highlighting the potential health complications related to female genital schistosomiasis (FGS) [47]. According to the WHO, genital schistosomiasis affects females and males, but the clinical signs and complications are much more prevalent in women. It is estimated that 56 million women and girls in the African region may be affected by FGS [48,49]. Furthermore, WHO recognizes existing complications in diagnosing FGS, pointing out that not every woman with schistosome eggs in their urine will have FGS, and not everyone with FGS has detectable eggs in their urine [48,50]. The WHO recognizes *S*. *haematobium* as the major cause of FGS, and most patients and health workers confuse the disease with infertility, cancer, and sexually transmitted infections (STIs) [16,48]. A retrospective study conducted in Tanzania also indicated an increase in the trend of schistosomiasis-related cancers [4] that might be confused with male and female genital schistosomiasis, hence necessitating the need for the urgent need for appropriate diagnostics.

#### Nature of samples used for diagnosing schistosomiasis

For improved sensitivity, WHO recommends using urine and stool samples to diagnose intestinal and urogenital schistosomiasis, respectively [2,9]. These samples are noninvasive to patients, but the odor they produce during diagnosis can concern health practitioners and reduce the frequency of disease monitoring. The major challenge for changing samples for diagnosing schistosomiasis is associated with invasiveness and low sensitivity when other samples, such as blood serum, dried blood samples (DBS), finger prick blood, and saliva, are used, depending on the test of choice [9,13,21,41,51].

For instance, in a study conducted by Fuss and colleagues [41], serum-based qPCR missed 4 out of 86 positive (4.6%), while DBS-qPCR failed to detect 55% of positive samples, and the detection performance varied based on infection intensities. In the same study, the authors reported that POC-CCA performed much better with detection sensitivity (96.6%) compared to serum-qPCR (89.3%) and DBS-qPCR (65.5%) for the 100 to 399 eggs per gram of KK confirmed stool samples. The explanation for the reduced sensitivity of NAATs-based blood serum, DBS, and saliva tests is likely that the amount of circulating DNA, which mainly originate from *Schistosoma* eggs, is lower in cell-free samples than in stool and urine samples [21,52]. For antibody and antigen-based assays, detecting antibodies requires blood samples, and CCA and CAA-based tests shows much better sensitivity using serum and urine [15,27,30,53,54]. Still, serum samples fit nicely with clinical settings where the use of serum is preferred over urine, as opposed to field surveillance, where people prefer to provide non-invasive samples. It is, therefore, clear that targeting higher sensitivity and the non-invasiveness nature of stool, and patient-friendly urine samples make them the 2 samples significant respective of the sample characteristics.

#### TPPs and REASSURED criteria for schistosomiasis diagnostics

In 2021, the WHO highlighted that most countries health ministries lack effective tools for monitoring and evaluating schistosomiasis control programs [9]. In the same report, WHO pointed out that the current gold standard approach limits the frequency of monitoring schistosomiasis due to the need for trained personnel, a microscope, and sometimes stool samples, which are only sometimes friendly to health worker practitioners. Therefore, WHO developed diagnostic TPPs to guide the development of new diagnostics to detect schistosomiasis prevalence reliably [9]. According to the WHO, the new TPPs aims to improve the specificity and sensitivity of the existing diagnostic methods and seeks to enable the detection of non-stool or venous blood samples such as saliva, finger stick blood, or urine.

The diagnostic TPPs considered existing challenges to meeting all REASSURED criteria [55,56], and therefore, the combination of 2 in vitro tests was proposed to assess the monitoring and evaluation of schistosomiasis control programs and determine whether transmission has been interrupted reliably in the community. In this case, both tests might be point-of-care based, or one screening point-of-care, and the confirmatory test, which might be laboratory-based and much more expensive. The exclusion of stool and venous samples in new diagnostic TPPs might make it challenging to get the expected sensitivity for intestinal schistosomiasis, as studies indicate that much more sensitivity can be attained by targeting *S*. *mansoni* biomarkers in stool or blood serum samples [2,41]. Nevertheless, urine, saliva, finger prick blood, and breath have good potential for new diagnostics.

### Towards the development of new diagnostics

#### Prospects for identifying new diagnostics lie in schistosome genomes and life cycle stages

**Fig 2:**
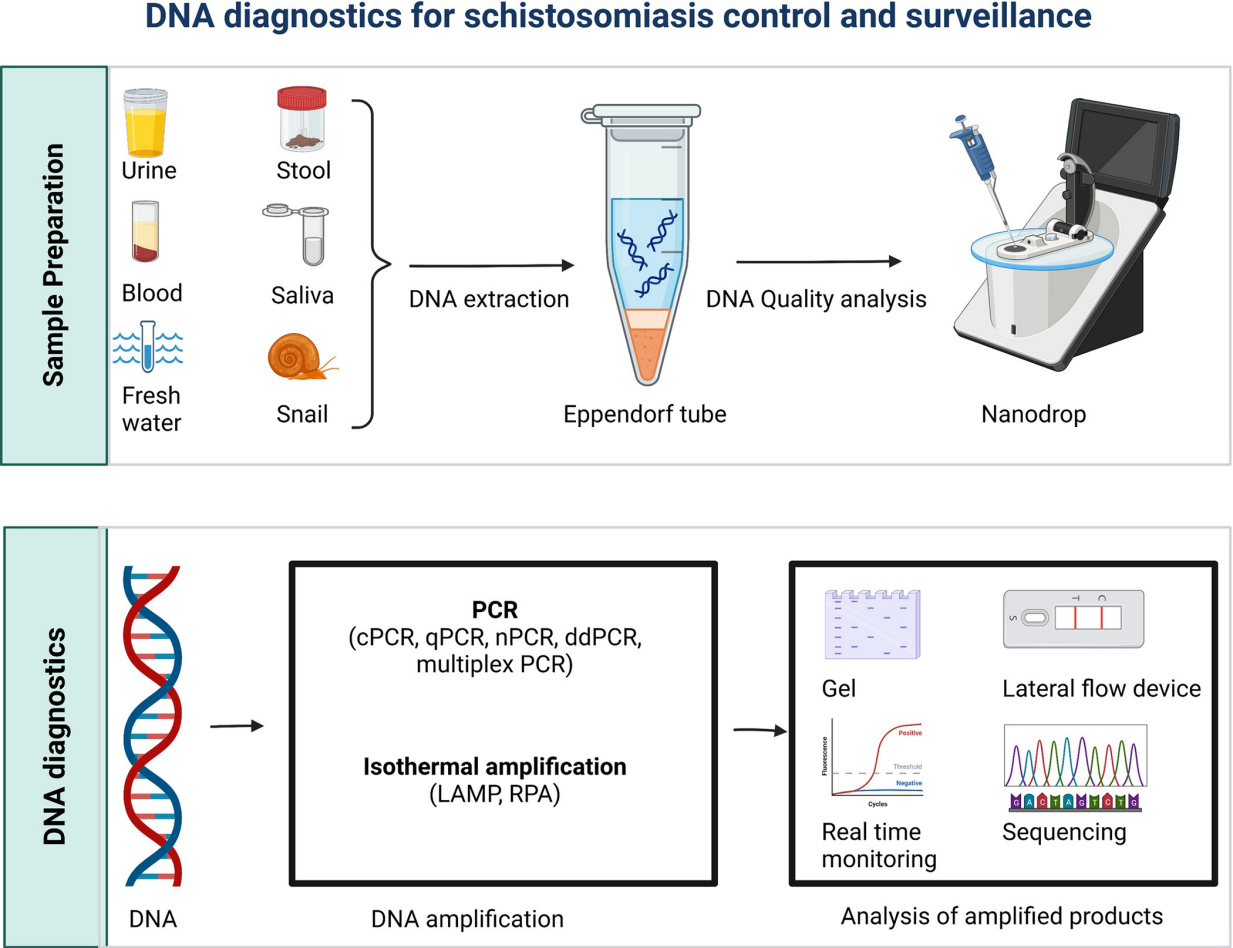
This figure shows the flow of DNA amplification-based approaches for diagnosing schistosomiasis. The process starts with DNA extraction followed by amplification and sequencing. Image created with BioRender.com.

The complete sequencing, assembly, and annotation of most schistosomes of medical importance have a great contribution to the development of new diagnostics (Fig 2).

The analysis shows that schistosome species are highly related, with their genomes made up of repeated sequences. The high synteny of most *Schistosoma* species [57,58] of medical importance causes the questionable specificity in most molecular-based assays developed before they were fully sequenced. For example, the in silico analysis, supported by DNA sequencing, has indicated the possibility for nucleic acid-based assays thought to be species-specific, to detect related species [59]. In addition, (WHO, 2022) through its guideline for controlling and eliminating human schistosomiasis, quoted, *“Further work is needed to characterize the sensitivity and specificity of immunological and molecular diagnostic tools for human schistosomiasis*.*”* Pointing out that molecular-based diagnostics need more sensitivity and specificity data [2]. Therefore, prior comparison of NAATs with KK or urine examination, the available genomic data for schistosomes and other helminths, freely available on databases would be used to further assess the specificity.

Schistosomes have 4 major stages: eggs, miracidia, cercaria, and adult schistosomes, and each stage can significantly contribute to the development of new diagnostics [1,60]. For instance, the POC-CCA and (UCP-LF)-CAA detect adult *Schistosoma*-based antigens, while the helmintex assay was designed to target *S*. *mansoni* eggs in the stool of an infected host [11,29,40]. Also, protein transcription in schistosomes is altered depending on the species, sex, and life cycle stages, and there is a study that characterized and described proteins in different life stages [57], and the variation in protein transcription has a significant effect on the performance of protein-based diagnostic assay. Targeting specific biomarker on a life cycle of schistosomes is therefore essential for targeting a single or multiple species and determining the stage and level of schistosomiasis.

#### Isothermal amplification methods

Portable isothermal amplification methods have been developed, including LAMP and RPA-based tests [33,43,45,61]. Unlike PCR, isothermal amplification assays do not require expensive equipment, and it is possible to integrate these technologies into lateral flow or CRISPR-based detection systems. For instance, a field-deployable ShDra1-RPA assay demonstrated the capacity to detect *S*. *haematobium* in as low as 10 eggs/ml urine [43], while recent finding reported RPA diagnostic assay integrated to CRISPR-cas13a detection system to offer better sensitivity and specificity comparable to qPCR, making an assay standing a chance for further validation and possibility to act as a gold standard in future [45]. The need for DNA extraction, cold chain transport, and storage of molecular reagents hinder the deployment of isothermal amplification tests to the field settings. However, recent innovations have enabled development of lyophilized isothermal amplification reagents to un-necessitate the need for cold chain transport system, and recent publication on extraction free LAMP assays further show the potential of isothermal amplification assays to be used for disease detection in limited resource settings [62,63].

#### Genome mining-based identification of identical multi-repeat sequences (IMRS)

Several studies have already reported increased false negative results when NAAT-based detection was used to detect schistosomes, and, in most cases, the reduced sensitivity occurs when targeting single to very few genome targets for the assay [10,31,44]. Computational approaches can be applied to identify highly repeated, identical genome sequences distributed over chromosomes of schistosomes, and this approach can improve the detection sensitivity of NAATs. In the malaria field, for instance, researchers have been able to use identical multi-repeat sequences (IMRS) to develop a highly sensitive assay, as an added advantage, could specifically amplify *Plasmodium falciparum* using a single temperature [64,65], offering a lower limit of detection of approximately 1 parasite/μl, and much more sensitivity than conventional PCR [64]. The IMRS-algorithm used to identify IMRS sequences in *P*. *falciparum* can also be used to identify repeated sequences of schistosomes to improve the detection sensitivity of NAATs-based assays to detect single or multiple *Schistosoma* species.

#### Nanobody-based diagnostics

The 2 major challenges for antibody-based diagnostics are the reduced specificity and the inability to differentiate the past and current schistosomiasis. Nanobody technology has been proposed to solve the abovementioned challenges [66,67], and it has been used to identify specific parasite antigens and nanobodies for several pathogens, including viruses, bacteria protozoa, and fungi [67]. Nanobodies are single chain proteins derived heavy chain of IgG antibodies. Unlike antibodies, nanobodies are small in size and relatively high thermostable, and their longer complementarity-determining region 3 (CDR3) allows tight binding of epitopes [68]. Nanobodies, which are easy to produce in large scale, could be key in development of highly sensitive, specific, and affordable diagnostics, which can be used in resource-limited settings such as sub-Saharan Africa. The use of the nanobody coupled with a lateral flow device has been proposed as the probable solution to the development of cost-effective, highly sensitive, specific, and rapid immunoassays for diagnosing peste des petits ruminants [69,70]. The use of nanobody technology remains to be investigated as an effective in diagnosis of schistosomiasis.

#### Portable sequencers

In most countries, schistosomiasis occurs as the co-infection of multiple *Schistosoma* species [1,3,71]. In 2017, one study proposed the combination of conventional PCR and sequencing for an assured specificity [59]. Furthermore, the manifestation of schistosomiasis is sometimes confused with other diseases, and hence sequencing could enable specific detection of multiple infections [16]. Third-generation sequencing using MinION (Oxford Nanopore Technologies, ONT) has proved the capacity to rapidly detect multiple viruses in real-time [72]. When connected to a computer, the platform has also been successfully used as a POC diagnostic to detect several pathogenic viruses, including Ebolavirus, Chikungunya virus, and hepatitis C virus [73,74]. Although this platform has not been investigated for most pathogens and is not commercially available as diagnostic tool in low-income countries, further research can offer solutions to the detection of multiple pathogens, including co-infection with schistosomiasis.

#### Imaging devices and AI-assisted diagnostics

One of the recent advancements in copro microscopic-based diagnosis is the development of portable and digitalized lab-on-a-disc devices, which offer high sensitivity in low transmission settings and can be used in remote areas [24,75,76]. Most of these tools were developed for the detection of soil-transmitted helminths, but they have already proven potential for diagnosing intestinal schistosomiasis. Studies have indicated the potential of the lab-on-a-disc devices to enable egg floatation, image capture and digitalized documentation, and hence enable the detection of soil-transmitted helminths, hookworms and *S*. *mansoni* [24,75]. Researchers have also managed to come up with a low cost Kubic FLOTAC microscope (KFM), which uses artificial intelligence for the recognition of helminth eggs, and on its added advantage, the device can be used without electricity in the laboratory and field settings [75]. The integration of AI on KFM enables the automated detection and counting of helminth eggs, and hence the operation of the device can be performed without any specific training. Further investigation of AI may play a great role in developing accurate and affordable diagnostics for detecting schistosome eggs and lesions caused by FGS.

#### Microfluidics devices

Lab-on-chip devices (LOC) called microfluidic are designed to allow the manipulation of small volumes of samples and reagents within microscale channels [77,78]. In 2016, a microfluidic device that allowed integrated trapping and isolation of parasite eggs was reported, which, after in-chip staining and fluorescence analysis, allowed microscopic imaging and enumeration of *S*. *haematobium* eggs [77]. The microscopic imaging allows high specificity comparable to microscopic examination, and the integrated trapping and isolation result in the concentration of schistosome eggs, which could potentially improve the detection sensitivity. The recent development of microfluidic devices allows low-cost detection of disease biomarkers, proteins, eggs, and DNA from pathogens and pathogenic microorganisms, and therefore, the incorporation of low-cost imaging systems such as cell phone, may potentially result in a POC diagnostics that meet much more REASSURED criteria to detect schistosomes in resources limited settings [55,56,77]. For DNA-based detection, the portable and disposable microfluidic cassette has already been evaluated to assess its potential to detect *S*. *mansoni*-positive blood samples, taking advantage of LAMP and the heating element integrated into a microfluidic device [79]. Further studies to eliminate the need for DNA extraction and the integration of AI might foster the adoption of DNA-based POC for schistosome detection.

## Conclusion and recommendation

Several assays have been developed for diagnosis of *Schistosoma* infections; however, sensitivity and specificity remain a major concern. There is an urgent need to re-evaluate, improve the existing assays, and identify new targets and assays for the detection of schistosomes that will adhere to the REASSURED criteria and meeting the TPP for schistosomiases elimination.

**Key learning points**

- Schistosomiasis is a neglected tropical disease with high prevalence in sub-Saharan Africa.
- *Schistosoma mansoni* and *S*. *haematobium* are the major causes of schistosomiasis, and diagnostic TPPs seek a low-cost, field-deployable test much more sensitive than existing approaches to detect multiple or single *Schistosoma* species.
- Artificial intelligence offers some field deployable diagnostics a high potential to attain much more REASSURED criteria or diagnostic TPPs for field-based monitoring and evaluating schistosomiasis control programs and determining the success of transmission interruption.

**Selected references**

World Health Organization [WHO]. Diagnostic target product profiles for monitoring, evaluation and surveillance of schistosomiasis control programmes. 2021;28. Available from: https://www.who.int/publications/i/item/9789240031104

World Health Organization [WHO]. WHO GUIDELINE on control and elimination of human schistosomiasis. 2022. 142 p.

Ogongo P, Nyakundi RK, Chege GK, Ochola L. The Road to Elimination: Current State of Schistosomiasis Research and Progress Towards the End Game. Front Immunol. 2022;13(May):1–23.

Graeff-Teixeira C, Favero V, Pascoal VF, de Souza RP, Rigo F de V, Agnese LHD, et al. Low specificity of point-of-care circulating cathodic antigen (POC-CCA) diagnostic test in a non-endemic area for schistosomiasis mansoni in Brazil. Acta Trop. 2021;217.

Hoermann J, Kuenzli E, Schaefer C, Paris DH, Bühler S, Odermatt P, et al. Performance of a rapid immuno-chromatographic test (Schistosoma ICT IgG-IgM) for detecting Schistosoma-specific antibodies in sera of endemic and non-endemic populations. PLoS Negl Trop Dis. 2022;16(5):1–11.
